# Robotic versus laparoscopic gastrectomy with splenic hilar lymph node dissection: short-term outcomes from a propensity score-matched study

**DOI:** 10.1007/s11701-026-03942-0

**Published:** 2026-09-12

**Authors:** Hironori Ohdaira, Junji Takahashi, Yosuke Igarashi, Norihiko Suzuki, Jungo Yasuda, Hiroya Enomoto, Eigoro Yamanouchi, Yutaka Suzuki

**Affiliations:** 1 Department of Surgery, International University of Health and Welfare Nasu Medical Center, Nasushiobara, Japan; 2 Department of Radiology, International University of Health and Welfare Nasu Medical Center, Nasushiobara, Japan

**Keywords:** Gastric cancer, Robotic gastrectomy, Laparoscopic gastrectomy, Splenic hilar lymph node dissection, Propensity score matching

## Abstract

Spleen-preserving dissection of the No. 10 (splenic hilar) nodal station is technically demanding, given the narrow working space bordered by terminal splenic vessels and the pancreatic tail. This study aimed to compare short-term outcomes of robotic and laparoscopic No. 10 dissection. We retrospectively compared 113 patients undergoing gastrectomy with No. 10 dissection (laparoscopic, *n* = 69; robotic, *n* = 44) at a single center (November 2008–March 2026). Propensity scores (age, sex, BMI, preoperative chemotherapy, clinical T4 status, nodal positivity) were used for 1:1 nearest-neighbor matching (0.2-SD logit caliper), yielding 35 pairs; paired outcomes were compared with Wilcoxon signed-rank and McNemar tests. In the matched cohort, robotic surgery had less blood loss (0 [IQR 0–20] vs. 30 [5–150] mL; *P* < 0.001), lower day-1 drain amylase (248 [161–481] vs. 593 [427–1113] IU/L; *P* = 0.001), and higher No. 10 nodal yield (1 [1–2] vs. 1 [1–1]; *P* = 0.009), with a trend toward longer operative time (404 [367–438] vs. 372 [333–402] min; *P* = 0.050). Total nodal yield, morbidity (overall 17.1% vs. 11.4%, *P* = 0.683; severe 14.3% vs. 11.4%, *P* = 1.000), and hospital stay (14 vs. 13 days; *P* = 0.339) did not differ; neither arm had splenic hilar fistula or No. 10 metastasis. Robotic gastrectomy was associated with less blood loss, lower pancreatic enzyme elevation, and higher No. 10 nodal yield than laparoscopy, without added morbidity or longer stay. Prospective, multicenter studies are needed to confirm and clarify the oncological rationale for routine No. 10 dissection.

**Trial registration:** University Hospital Medical Information Network (UMIN000032895).

**Supplementary Information:**

The online version contains supplementary material available at https://doi.org/10.1007/s11701-026-03942-0.

## Introduction

Among gastrointestinal cancers, gastric adenocarcinoma remains one of the most common, with mortality that is especially high across East Asia [1]. Curative-intent treatment centers on radical gastrectomy with systematic lymph node dissection, and how thoroughly the nodal basins are cleared has a direct bearing on oncological outcome. For proximal tumors that reach the greater curvature, the splenic hilar (No. 10) basin becomes clinically relevant, since historical surgical series report metastasis rates there of 9%–26% [2]—high enough to justify deliberate dissection in this subgroup. For many years this risk was addressed simply by removing the spleen together with the stomach during total gastrectomy, the most direct way of clearing the station.

Whether prophylactic splenectomy actually prolongs survival was settled by the JCOG0110 phase III trial, conducted in patients with proximal gastric cancer whose tumors spared the greater curvature. Splenectomy conferred no survival advantage over spleen preservation and was instead associated with more intraoperative bleeding and more postoperative complications [3]; elective splenectomy in this setting is therefore no longer recommended. The question is less settled, however, for tumors that do involve the greater curvature, since that is precisely the anatomical setting in which No. 10 metastasis is most likely—here, dissection of the hilar basin beyond the standard D2 field is still practiced at experienced centers. In their review of the evidence, Kinoshita and Okayama argued against prophylactic splenectomy when the greater curvature is spared, noted that a survival benefit from No. 10 dissection in greater-curvature-involving disease is suggested by retrospective series but not yet proven prospectively, and predicted that minimally invasive spleen-preserving techniques will increasingly replace splenectomy in this indication [4].

Consistent with these guideline positions [2, 4], our institutional policy favors spleen-preserving hilar dissection whenever the vascular anatomy and tumor extent allow it, reserving splenectomy or distal pancreatectomy for cases where safe preservation is not intraoperatively feasible. Laparoscopic execution of this step became established in the years after the first report of laparoscopic gastrectomy in 1994 [5], with subsequent series from several groups documenting its feasibility [6–8]. More recently, robotic platforms have offered an alternative: magnified three-dimensional visualization, wristed instruments with several degrees of freedom, and tremor filtration may together permit finer dissection within the tight space surrounding the splenic hilar vessels [9, 10]. Robotic gastrectomy volumes in Japan rose sharply once national health insurance began covering the procedure in April 2018 [11, 12].

Despite this accumulating experience, few studies have directly compared laparoscopic and robotic spleen-preserving No. 10 dissection, and data on how often and why surgeons convert to splenectomy or pancreatectomy remain limited. We therefore conducted this single-center, propensity-score-matched analysis to compare short-term surgical performance and oncological surrogate outcomes between the two approaches.

## Methods

### Study design and patients

**Fig. 1:**
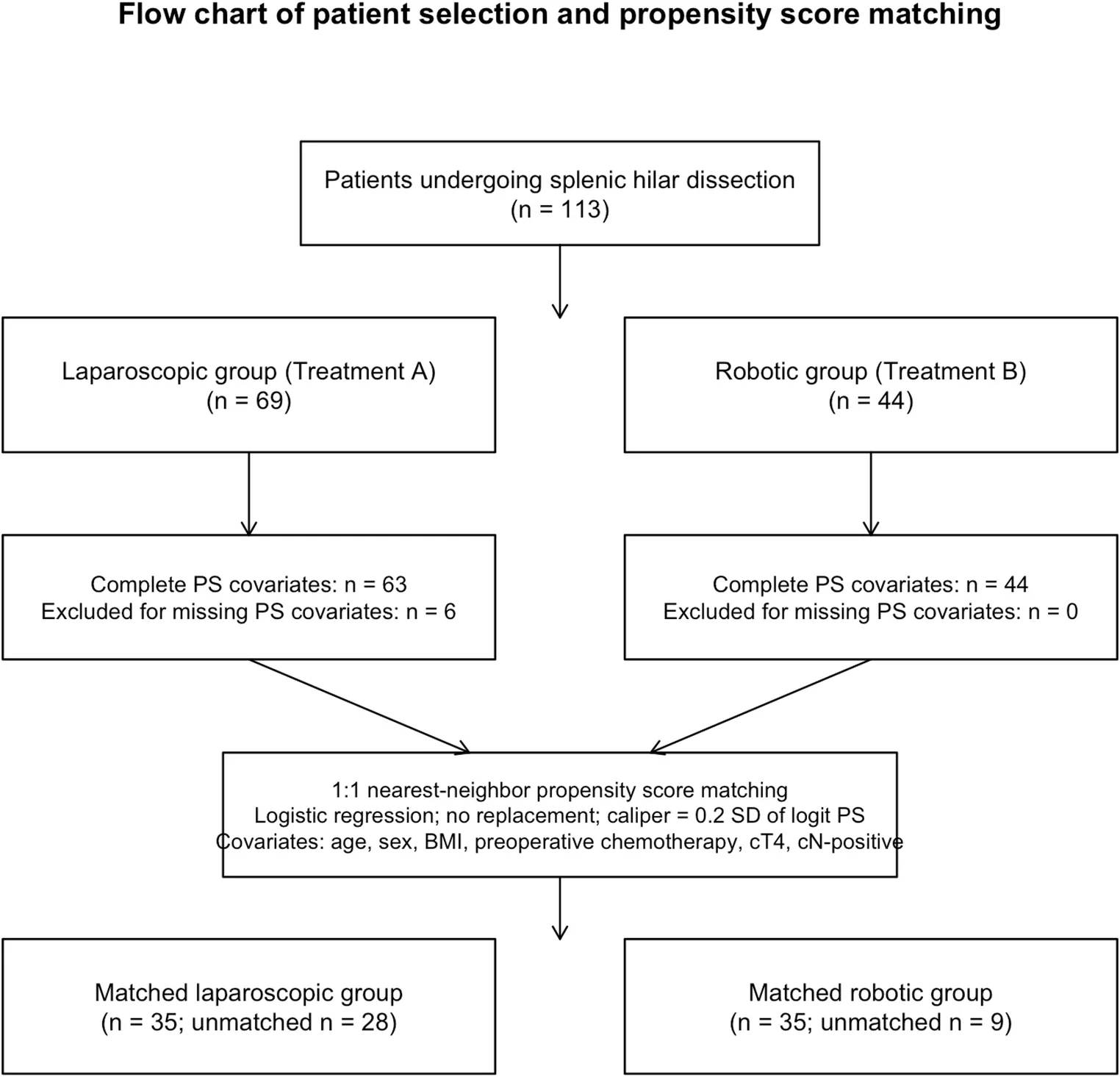
Flowchart of patient selection and propensity score matching. The propensity model incorporated age, sex, BMI, preoperative chemotherapy, clinical T4 status, and nodal positivity (cN+). Non-replacement 1:1 nearest-neighbor matching on the logit propensity score (0.2-SD caliper) produced 35 balanced pairs. BMI, body mass index; PS, propensity score

This retrospective study, conducted in the Department of Surgery at International University of Health and Welfare Nasu Medical Center, Japan, included all consecutive patients who underwent gastrectomy with No. 10 lymph node dissection for greater-curvature-involving gastric cancer between November 2008 and March 2026. Of the 113 identified patients, 69 underwent the laparoscopic procedure (November 2008–August 2019) and 44 underwent the robotic procedure (July 2015–March 2026). The patient-selection and matching workflow is summarized in Fig. 1. Institutional ethics board approval was obtained, and the study was conducted according to the principles of the Declaration of Helsinki; further ethics and consent details are given under Declarations.

## Surgical policy and indications

For proximal tumors involving the greater curvature, our default approach was dissection anterior to the splenic hilar vessels with the spleen left in place. We converted to splenectomy or distal pancreatectomy intraoperatively whenever bulky or confluent nodal disease, direct tumor extension, or vascular encasement made spleen preservation unsafe; this intraoperative judgment rested with the operating surgeon alone.

## Technique of spleen-preserving splenic hilar dissection

The omental bursa was opened first, and the left gastroepiploic artery was divided at its origin. Dissection then continued within the loose, relatively avascular plane between the perivascular neurovascular bundle and the adjacent fat, dividing the short gastric vessels one by one as the plane was extended along the distal splenic artery toward the upper pole of the spleen. If exposure remained inadequate despite retraction, we transected the abdominal esophagus early, before completing the hilar dissection, to gain additional working space.

## Splenectomy and distal pancreatectomy

In cases requiring splenectomy, the pancreatic body was mobilized while attempting to spare the caudal pancreatic vessels whenever the anatomy allowed. Where distal pancreatectomy was additionally needed, the pancreatic parenchyma was divided using a linear stapler with an extra-thick (black) cartridge.

## Laparoscopic approach

For laparoscopic cases, exposure of the splenic hilum depended on the bedside assistant, who lifted the stomach cephalad while retracting the transverse mesocolon caudally; an ultrasonic coagulating shears provided both cutting and hemostasis throughout. Because the ports lie relatively far from the hilar target, maintaining consistently good assistant retraction was essential to the dissection. A representative laparoscopic case is included as Online Resource 1.

### Robotic approach

Robotic cases were performed exclusively with a da Vinci system. Hemostasis around the main splenic vessels was achieved with fenestrated bipolar forceps on the first arm, while the third-arm monopolar curved scissors handled tissue division. Individual short gastric vessels were secured using Maryland bipolar forceps in soft-coagulation mode (effect 7, 80-W ceiling). Fourth-arm retraction, supplemented by traction sutures when needed, kept the hilum exposed throughout and largely eliminated the need for bedside-assistant retraction. A representative robotic case is included as Online Resource 2.

## Outcomes

Baseline variables collected for each patient included age, sex, BMI, prior systemic chemotherapy, ASA physical-status class, clinical and pathological T/N categories and corresponding overall stage, tumor histology, and maximum tumor diameter. Primary short-term endpoints were operative time, intraoperative blood loss, total and No. 10-specific nodal yield, postoperative day-1 drain amylase, and splenic hilar fistula—diagnosed from drain fluid characteristics together with CT findings. Postoperative complications were graded by the Clavien–Dindo system [13], with overall morbidity defined as grade ≥ II and severe morbidity as grade ≥IIIa. Secondary endpoints were No. 10 nodal metastasis, postoperative hospital stay, and in-hospital death.

## Propensity score matching

Because treatment was not randomly allocated, we used propensity score matching to limit confounding. A logistic regression model estimated each patient’s probability of undergoing robotic rather than laparoscopic surgery from six covariates: age, sex, BMI, preoperative chemotherapy, clinical T4 disease (T4 vs. non-T4), and clinical node positivity (cN + vs. cN0). For the propensity model only, T and N categories were collapsed into binary variables to avoid instability from sparse cells; the complete ordinal staging appears in Table 1. Complete covariate data were available for 107 of the 113 patients (63 laparoscopic, 44 robotic), who formed the matching pool. We applied 1:1 nearest-neighbor matching without replacement on the logit-transformed propensity score, using a caliper of 0.2 standard deviations [14, 15]; this produced 35 matched pairs. Balance before and after matching was assessed with the standardized mean difference (SMD).

**Table 1:** Patient characteristics of the propensity-score-matching-eligible cohort (*n* = 107)

Variables	Laparoscopic (*n* = 63)	Robotic (*n* = 44)	*P* value
**Demographics**			
Age, median (years)	67.0	68.5	0.070
Male, n (%)	45 (65.2)	31 (70.5)	0.682
BMI, median [kg/m² (IQR)]	22.3 (20.8–23.9)	22.6 (21.2–24.8)	0.253
**Preoperative factors**			
Neoadjuvant chemotherapy, n (%)	4 (6.3)	4 (9.1)	1.000
ASA-PS			0.226
1, n (%)	36 (57.1)	25 (56.8)	
2, n (%)	27 (42.9)	17 (38.6)	
3, n (%)	0 (0.0)	2 (4.5)	
Clinical stage (UICC 8th)			0.131
Stage 1, n (%)	31 (49.2)	14 (31.8)	
Stage 2, n (%)	10 (15.9)	10 (22.7)	
Stage 3, n (%)	22 (34.9)	18 (40.9)	
Stage 4, n (%)	0 (0.0)	2 (4.5)	
**Pathological findings**			
Histological type			0.420
Tubular adenocarcinoma, n (%)	29 (46.0)	27 (61.4)	
Poorly differentiated, n (%)	22 (34.9)	13 (29.5)	
Signet-ring cell, n (%)	8 (12.7)	3 (6.8)	
Others, n (%)	4 (6.3)	1 (2.3)	
pT category			0.300
T1, n (%)	32 (50.8)	16 (36.4)	
T2, n (%)	5 (7.9)	2 (4.5)	
T3, n (%)	12 (19.0)	14 (31.8)	
T4, n (%)	14 (22.2)	12 (27.3)	
pN category			0.965
N0, n (%)	36 (57.1)	25 (56.8)	
N1, n (%)	9 (14.3)	7 (15.9)	
N2, n (%)	6 (9.5)	5 (11.4)	
N3, n (%)	12 (19.0)	7 (15.9)	
R0 resection, n (%)	63 (100.0)	44 (100.0)	1.000

IQR, interquartile range; BMI, body mass index; ASA-PS, American Society of Anesthesiologists physical status; UICC, Union for International Cancer Control. Six laparoscopic patients with missing propensity score covariates were excluded from this table (total laparoscopic cohort *n* = 69)

*P *values: Mann-Whitney U test for continuous variables; Fisher’s exact test for categorical variables

### Statistical analysis

Continuous variables are summarized as median (interquartile range) and categorical variables as counts (percentages). In the unmatched cohort, group comparisons used the Mann–Whitney U test for continuous variables and Fisher’s exact test for categorical variables; in the matched cohort, within-pair comparisons used the Wilcoxon signed-rank test for continuous outcomes and McNemar’s test for categorical outcomes, consistent with standard practice for matched data. All tests were two-sided, with *P* < 0.05 considered statistically significant. To visualize the evolution of operative efficiency over time, cases within each arm were ordered chronologically and a five-case moving average of operative time was calculated—a method commonly used to depict surgical learning curves [16, 17], including during robotic program introduction [18]; cases missing operative-time data were excluded from this specific analysis. All analyses were performed in R, version 4.6.0 (R Foundation for Statistical Computing, Vienna, Austria).

## Results

### Patient selection and baseline characteristics

Among the 113 enrolled patients, 69 had laparoscopic and 44 had robotic surgery. Missing covariate data excluded six laparoscopic patients from propensity estimation, leaving 63 laparoscopic and 44 robotic patients eligible for matching; 35 pairs (70 patients) were ultimately matched (Fig. 1). Before matching, median age (67.0 vs. 68.5 years; *P* = 0.070) and the male proportion (65.2% vs. 70.5%; *P* = 0.682) were similar between the laparoscopic and robotic groups, as were BMI, preoperative chemotherapy use, ASA-PS, clinical/pathological stage, and histology (Table 1); all patients in both groups achieved R0 resection (Table 1). After matching, four of the five model covariates—age, sex, BMI, and cT4 status—achieved SMDs ≤ 0.10, while cN positivity had a slightly higher SMD of 0.114; some imbalance remained in the more granular cN and clinical-stage subcategories (Table 1, Supplementary Figure S1).

### Operative and short-term postoperative outcomes

Before matching, robotic cases took longer in the operating room than laparoscopic cases (404.5 [IQR 372.0–438.0] vs. 379.0 [340.0–434.0] min; *P* = 0.038), but had less blood loss (0 [0–20] vs. 50 [5–150] mL; *P* < 0.001) and a lower drain amylase level (285 [194.5–649.0] vs. 593 [405.5–1300.5] IU/L; *P* < 0.001). Overall nodal yield was similar between groups, whereas No. 10-specific yield favored the robotic approach (1 [1–2] vs. 1 [1–1]; *P* < 0.001). Hospital stay was longer in the unmatched robotic cohort (14.5 [11–23] vs. 10 [8–16] days; *P* < 0.001).

These patterns largely persisted after matching: the robotic group again had less blood loss (0 [0–20] vs. 30 [5–150] mL; *P* < 0.001), lower drain amylase (248 [161–481] vs. 593 [427–1113] IU/L; *P* = 0.001), and a higher No. 10 nodal yield (1 [1–2] vs. 1 [1–1]; *P* = 0.009). Operative time was still somewhat longer with the robotic approach, though the difference was only borderline significant (404 [367–438] vs. 372 [333–402] min; *P* = 0.050). Total lymph node retrieval (37 [29–45] vs. 34 [25–40]; *P* = 0.339) and hospital stay (14 [10–19] vs. 13 [9–20] days; *P* = 0.339) no longer differed between the matched groups (Table 2).

**Table 2:** Operative and short-term postoperative outcomes

Variables	Total cohort			Matched cohort		
	Lap (*n* = 69)	Robotic (*n* = 44)	*P*	Lap (*n* = 35)	Robotic (*n* = 35)	*P*
**Intraoperative outcomes**						
Operative time, min	379.0 (340.0–434.0)	404.5 (372.0–438.0)	0.038	372 (333–402)	404 (367–438)	0.050
Blood loss, mL	50 (5–150)	0 (0–20)	< 0.001	30 (5–150)	0 (0–20)	< 0.001
Drain amylase, IU/L	593 (405.5–1300.5)	285 (194.5–649.0)	< 0.001	593 (427–1113)	248 (161–481)	0.001
No. 10 lymph nodes retrieved	1 (1–1)	1 (1–2)	< 0.001	1 (1–1)	1 (1–2)	0.009
Total lymph nodes retrieved	—	—	—	34 (25–40)	37 (29–45)	0.339
**Postoperative outcomes**						
Postoperative hospital stay, days	10 (8–16)	14.5 (11–23)	< 0.001	13 (9–20)	14 (10–19)	0.339
Overall morbidity, Grade II+, n (%)	—	—	—	4 (11.4)	6 (17.1)	0.683
Severe morbidity, Grade IIIa+, n (%)	—	—	—	4 (11.4)	5 (14.3)	1.000
Splenic hilar fistula, n (%)	—	—	—	0 (0.0)	0 (0.0)	—
No. 10 lymph node metastasis, n (%)	—	—	—	0 (0.0)	0 (0.0)	—
In-hospital mortality, n (%)	—	—	—	0 (0.0)	0 (0.0)	—

Lap, laparoscopic group. Complications graded per Clavien-Dindo classification; overall morbidity = Grade II or higher; severe morbidity = Grade IIIa or higher.

Total cohort P values: Mann-Whitney U test (continuous) / Fisher’s exact test (categorical).

Matched cohort P values: paired Wilcoxon signed-rank test (continuous) / McNemar’s test (binary), reflecting the paired structure after 1:1 matching.

### Postoperative morbidity and mortality

Grade ≥ II complications by the Clavien–Dindo system occurred in 11.4% of laparoscopic and 17.1% of robotic patients in the matched cohort (*P* = 0.683), and grade ≥IIIa events in 11.4% and 14.3%, respectively (*P* = 1.000). No evaluable patient developed a splenic hilar fistula or No. 10 nodal metastasis, no patient required readmission or reinsertion of a drain for intra-abdominal fluid collection, and there were no in-hospital deaths among matched patients. Within the full unmatched cohort, a single laparoscopic patient experienced a grade V complication.

### Moving-average analysis of operative time

**Fig. 2:**
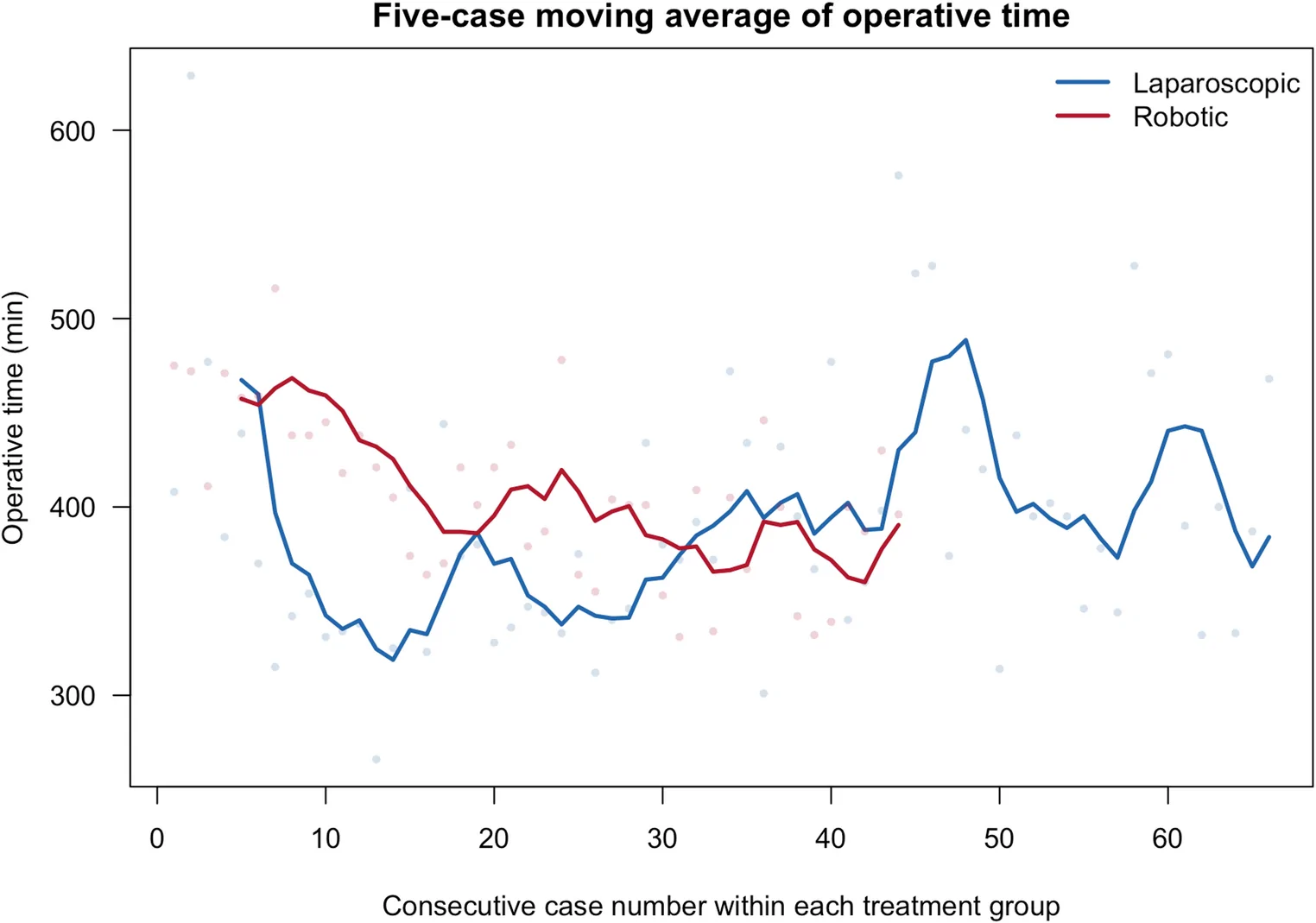
Chronological five-case moving averages of operative time for each treatment arm. Individual case durations appear as translucent points, with moving-average lines overlaid to show the smoothed trend; cases lacking operative-time data were excluded

Plotting the five-case moving average of operative time showed a steady decline from the early robotic experience toward a stable plateau, in contrast to the laparoscopic arm, whose moving average fluctuated irregularly across the study period without a clear trend (Fig. 2). This was a descriptive analysis only; it was not intended to define a formal inflection point or to estimate the case volume required to complete a learning curve.

## Discussion

In this propensity-matched comparison, robotic surgery was associated with several measurable technical differences relative to laparoscopy for No. 10 dissection: less intraoperative bleeding, a smaller day-1 rise in drain pancreatic enzymes, and a greater number of retrieved hilar nodes. A plausible mechanism underlying these associations is the greater dexterity of wristed robotic instruments combined with the stable, magnified stereoscopic view they provide within the tight anatomical confines around the splenic hilar vessels [9, 10]. Importantly, none of these differences appeared to come at a cost: morbidity (overall and severe), total nodal yield, and hospital stay were statistically equivalent between groups, and neither cohort experienced a splenic hilar fistula—findings consistent with organ-preserving hilar dissection being performed safely at experienced centers, regardless of platform.

No. 10 dissection is technically demanding largely because the target nodes sit immediately adjacent to terminal branches of the splenic artery and vein and to the pancreatic tail, leaving little margin for error between incomplete clearance and vascular or pancreatic injury. The seven degrees of freedom and motion scaling built into robotic instruments are well suited to this constraint [9, 10]. In one of the larger published robotic series, Yang et al. reported 92 patients undergoing robotic spleen-preserving hilar dissection during total gastrectomy, with a median No. 10 yield of 1.9 nodes, 16.3% overall morbidity, and no pancreatic fistulas [9]—broadly consistent with what we observed in our own robotic arm. Taken together, the lower blood loss, lower amylase elevation, and higher nodal yield we observed are consistent with a more controlled, tissue-selective dissection plane with the robotic platform, although we acknowledge that drain amylase is only an indirect marker of pancreatic injury.

These findings echo a multicenter randomized trial in which robotic gastrectomy was linked to lower overall rates of grade ≥ II and grade ≥IIIa complications than laparoscopy, although that trial found no specific advantage for intra-abdominal infectious complications [21]. Read together, the two datasets suggest that robotic benefit is not uniform but scales with how technically demanding a given operative step is—the confined, vessel-dense hilar field, where rigid laparoscopic instruments are at their greatest geometric disadvantage, may be exactly where articulated robotic arms provide the most clinical benefit.

JCOG0110 demonstrated that, for proximal tumors sparing the greater curvature, preserving the spleen is oncologically equivalent to splenectomy while lowering perioperative morbidity [3]. Reflecting this evidence, current Japanese treatment guidelines restrict extended (beyond-D2) No. 10 dissection to tumors that clearly involve the greater curvature and otherwise favor spleen preservation whenever technically possible [2]. In keeping with this conservative approach, we found no No. 10 metastasis among the 108 evaluable patients in our series, consistent with the generally low background rate of hilar nodal disease and supportive of an organ-preserving strategy as the default. The apparent precision of the robotic platform in this region suggests that careful, image-based patient selection combined with meticulous hilar clearance may help achieve oncological adequacy without added surgical trauma.

Robotic operative times were somewhat longer overall, but the five-case moving average showed clear improvement during the early period, settling toward a plateau thereafter. Similar moving-average patterns reported in other robotic gastrectomy series have shown operative-time stabilization after roughly 100–150 cases—often more quickly than the corresponding laparoscopic learning curve [18, 19]—while a large multicenter cumulative-sum analysis of robotic distal and total gastrectomy placed the learning-curve inflection point at around 20–22 cases [20]. Our own trajectory of early prolongation followed by a plateau fits this general pattern, suggesting that the current operative-time gap should narrow further as robotic case volume at our institution continues to grow.

Several limitations should be noted. This was a retrospective, single-center study spanning 18 years, during which laparoscopic cases clustered in the earlier period (2008–2019) and robotic cases in the later period (2015–2026); secular changes in perioperative care, enhanced-recovery practice, and adjuvant treatment over this interval are potential confounders that propensity matching cannot fully remove. With 35 matched pairs, the study had reasonable power to detect the observed differences in blood loss, drain amylase, and No. 10 yield, but was underpowered for rare adverse events. Drain amylase beyond postoperative day 1 could not be analyzed because of substantial missing values, limiting our ability to characterize the full time-course of pancreatic enzyme elevation and its relationship to clinically evident pancreatic fistula. Some post-matching imbalance remained in detailed nodal and staging subcategories, and certain intraoperative variables were not fully standardized—limitations inherent to a retrospective, single-institution design. Prospective, multicenter studies with pre-specified protocols and contemporaneous comparison groups will be needed to confirm and extend these preliminary observations.

In summary, robotic-assisted spleen-preserving splenic hilar dissection was associated with lower blood loss, less pancreatic enzyme release, and greater No. 10 nodal harvest than the laparoscopic approach, while safety and short-term oncological indicators remained equivalent between platforms. These preliminary, hypothesis-generating findings suggest the robotic platform as a technically promising minimally invasive option for this anatomically demanding nodal station, pending confirmation of long-term oncological efficacy and refinement of patient-selection criteria for No. 10 dissection through well-designed, prospective, multicenter trials.

## Supplementary Information

Below is the link to the electronic supplementary material.

**Online Resource 1** Laparoscopic spleen-preserving splenic hilar lymph node dissection. This video shows exposure of the hilar field via assistant-controlled retraction of the stomach and transverse mesocolon, division of the left gastroepiploic artery at its origin, stepwise development of the loose areolar plane between the perivascular neurovascular structures and the surrounding lymphofatty tissue with an ultrasonic shears device (LCS), and sequential individual ligation of the short gastric vessels and distal splenic arterial branches up to the splenic upper pole. (MP4)

**Online Resource 2** Robotic spleen-preserving splenic hilar lymph node dissection using the da Vinci system. This video demonstrates bipolar coagulation near the main splenic vessel trunk with the first-arm fenestrated bipolar forceps, dissection of the tissue plane with the third-arm monopolar curved scissors, individual short gastric vessel ligation using Maryland bipolar forceps in soft-coagulation mode (effect 7, 80-W ceiling), and continuous hilar exposure maintained by the fourth robotic arm without active bedside-assistant retraction. (MP4)

**Supplementary Figure S1** Love plot showing absolute standardized mean differences (SMD) for each covariate before (open circles, unmatched) and after (closed triangles, matched) propensity score matching. The dashed vertical line indicates the pre-specified balance threshold of SMD = 0.10. BMI, body mass index; cT4, clinical T4 disease; cN+, clinically node-positive status; PS, propensity score.

## Data Availability

No datasets were generated or analysed during the current study.
