# Supplementary material for: Robotic versus laparoscopic gastrectomy with splenic hilar lymph node dissection: short-term outcomes from a propensity score-matched study
Source: J Robot Surg. 2026 Sep 12;20(1):982. doi: 10.1007/s11701-026-03942-0 (PMC13570898; doi:10.1007/s11701-026-03942-0)
Supplement: Supplementary file 1 [file 11701_2026_3942_MOESM1_ESM.docx]

**Supplementary Figure S1. Covariate balance before and after propensity score matching**

*Love plot showing absolute standardized mean differences before and after propensity score matching. The dashed vertical line indicates the prespecified balance threshold of 0.10. PS, propensity score; BMI, body mass index; cT4, clinical T4 disease; cN-positive, clinically node-positive disease.*
